# Two Cases in Which the Pneumatic Aspirator Was Employed Successfully

**Published:** 1874-06

**Authors:** E. W. Lee

**Affiliations:** Chicago


A MONTHLY RECORD OF

*Medicine, Surgery and the Collateral Sciences.*

EDITED BY J. ADAMS ALLEN, M.D., LL.D.; AND WALTER HAY, M.D.

VOL. XXXI.—JUNE, 1874.—No. 6.

### Original Communications.

ARTICLE I.—*Two Cases in which the Pneumatic Aspirator was Employed Successfully.* By E. W. LEE, M.D., Chicago.

CASE 1. G. M., aged 74 years, called upon me early on the morning of February 5th, complaining of inability to urinate. A few questions elicited evidences of chronic prostatic enlargement, which was confirmed by a digital examination, the gland being of great size, indurated, but quite insensitive, pressure being freely borne without pain.

I at once attempted to empty the bladder by catheterism, but after repeated efforts with various sizes of instruments, was reluctantly obliged to desist. I then resorted to hip baths, injections per rectum of warm water, with opium, etc., internally. A second and repeated attempts to introduce the catheter at intervals until the afternoon of the 6th, were invariably unsuccessful. Up to this time the kidneys were not secreting actively, hence there was no painful distention; but slight symptoms of coma ensuing, it became imperative to at once empty the bladder and promote free action of the kidneys. As I could not introduce the catheter, and puncturing the viscus through the rectum was contra-indicated by age and general condition, I determined to use my pneumatic aspirator, which was done with the most gratifying results. A medium sized needle of the aspirator being introduced immediately above the pubis, allowed the escape of thirty ounces of turbid and bloody urine. The operation was repeated twice on the 7th, thirty-five and forty-two ounces respectively being discharged, more nearly normal in quality. On the morning of the 8th I succeeded in carrying a No. 8 catheter into the bladder with comparative facility, and on the 9th the patient passed the instrument himself. There was not the slightest untoward symptom from the punctures of the bladder, and the man made a good recovery.

CASE 2. I was called, on the evening of the 22d of March, to see T. K., male, aged 30 years. He informed me that he had suffered for years from hernia, and that hitherto he could readily reduce it, but was now unable to do so. On examination I found a large scrotal hernia, strangulated. The man was in great pain, vomiting freely; skin cold and clammy; pulse fifty beats per minute, and weak; anxious expression of countenance.

I injected half a grain morph. sulph. hypodermically, with marked benefit, the pulse getting firmer and the skin warm and dry, pain also being removed to a great extent. I now had the patient anæsthetized, and having applied taxis until I became satisfied that it could not succeed, I introduced the finest needle of the aspirator, and drew off seven ounces of rather thick, bloody serum. Withdrawing the instrument I introduced one of larger calibre, which had the effect of liberating a quantity of air. These procedures reduced the volume of the tumor considerably, when, to my great satisfaction, with very little effort, the intestine was returned to the cavity to which it belonged. There was some tympanitis and tenderness of the abdomen for a couple of days, but the man made a good recovery.

